# Nuclear pore density controls heterochromatin reorganization during senescence

**DOI:** 10.1101/gad.321117.118

**Published:** 2019-02-01

**Authors:** Charlene Boumendil, Priya Hari, Karl C.F. Olsen, Juan Carlos Acosta, Wendy A. Bickmore

**Affiliations:** 1 Medical Research Council Human Genetics Unit, Institute of Genetics and Molecular Medicine, University of Edinburgh, Edinburgh EH4 2XU, United Kingdom;; 2 Cancer Research UK Edinburgh Centre, Institute of Genetics and Molecular Medicine, University of Edinburgh, Edinburgh EH4 2XU, United Kingdom

**Keywords:** inflammation, nuclear organization, nuclear pore, senescence

## Abstract

During oncogene-induced senescence (OIS), heterochromatin is lost from the nuclear periphery and forms internal senescence-associated heterochromatin foci (SAHFs). We show that an increased nuclear pore density during OIS is responsible for SAHF formation. In particular, the nucleoporin TPR is necessary for both formation and maintenance of SAHFs. Loss of SAHFs does not affect cell cycle arrest but abrogates the senescence-associated secretory phenotype—a program of inflammatory cytokine gene activation. Our results uncover a previously unknown role of nuclear pores in heterochromatin reorganization in mammalian nuclei and demonstrate the importance of heterochromatin organization for a specific gene activation program.

## Abstract (precis)

Here, Boumendil et al. show that an increased nuclear pore density during oncogene-induced senescence (OIS) is responsible for senescence-associated heterochromatin focus (SAHF) formation. They show that the nucleoporin TPR is necessary for both formation and maintenance of SAHF, identify a previously unknown role of nuclear pores in heterochromatin reorganization in mammalian nuclei, and demonstrate the importance of heterochromatin organization for a specific gene activation program.

Three-dimensional (3D) genome organization is governed by a combination of polymer biophysics and biochemical interactions, including local chromatin compaction, long-range chromatin interactions, and interactions with nuclear structures. One such structure is the nuclear lamina (NL), which coats the inner nuclear membrane and is composed of lamins and membrane-associated proteins, such as Lamin B receptor (LBR). Electron microscopy (EM) reveals large blocks of heterochromatin associated with the nuclear periphery (Capelson and Hetzer 2009), and mapping genome interactions with laminB1 identifies >1000 lamina-associated domains (LADs). LADs are associated with heterochromatic histone marks (H3K27me3 or H3K9me3) (Guelen et al. 2008). Altered NL composition in the photoreceptors of nocturnal mammals leads to the loss of heterochromatin from the nuclear periphery and its accumulation at the center of the nucleus (Solovei et al. 2013).

Another situation in which there is a dramatic reorganization of heterochromatin is in oncogene-induced senescence (OIS)—a cell cycle arrest program triggered by oncogenic signaling. OIS cells undergo striking chromatin reorganization with loss of heterochromatin and constitutive LADs (Lenain et al. 2017) from the nuclear periphery and the appearance of internal senescence-associated heterochromatin foci (SAHFs). SAHFs appear consecutive to cell cycle arrest and are not observed in nontransformed replicating cells (Narita et al. 2003). SAHF formation results from a reorganization of pre-existing heterochromatin—regions decorated with H3K9me3, H3K27me3, macroH2a, and HP1α,β,γ—rather than de novo heterochromatin formation on new genomic regions (Narita et al. 2003; Zhang et al. 2005; Chandra et al. 2012; Sadaie et al. 2013). Known factors implicated in SAHF formation include activation of the pRB pathway (Narita et al. 2003), certain chromatin-associated nonhistone proteins (Narita et al. 2006), and the histone chaperones HIRA and Asf1a (Zhang et al. 2005, 2007). The NL has also been implicated in SAHF formation: LaminB1 and LBR expression are decreased in OIS, and their experimental depletion can facilitate, but is not sufficient for, SAHF formation (Sadaie et al. 2013; Lukášová et al. 2017).

The nuclear envelope is perforated by nuclear pores that control transport between the cytoplasm and nucleus. The nuclear pore complex (NPC) is a large transmembrane complex consisting of ∼30 proteins called nucleoporins (Fig. 1A; Kim et al. 2018). In contrast to the adjacent NL, EM and superresolution light microscopy show that the nuclear area underneath nuclear pores is devoid of heterochromatin (Schermelleh et al. 2008; Capelson and Hetzer 2009), and nuclear pore density in different neurons and glial cell types from the rat cerebellar cortex anticorrelates with compact chromatin (Garcia-Segura et al. 1989). The nucleoporin TPR has been shown to be responsible for heterochromatin exclusion zones at the NPC (Krull et al. 2010).

**Figure 1.**
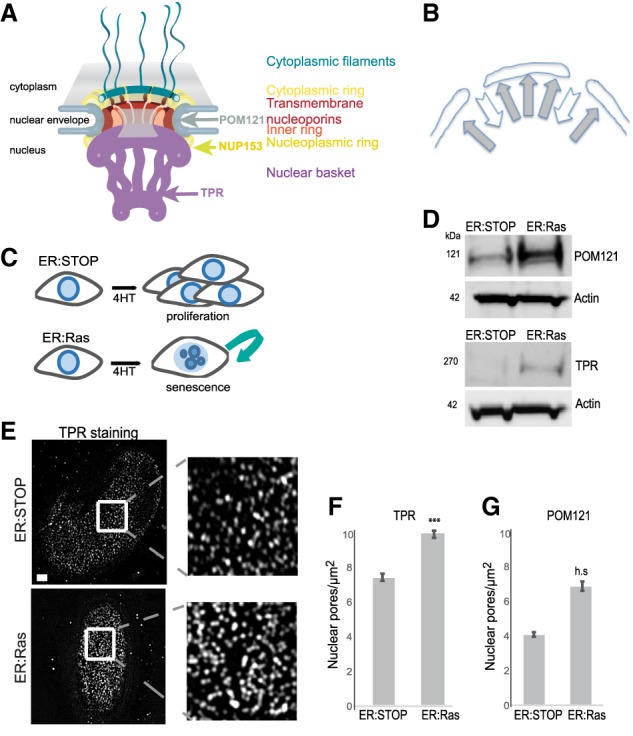
Nuclear pore density increases in OIS. (*A*) Model of the NPC showing the position of TPR, NUP153, and POM121. (*B*) Schematic showing the balance of forces attracting heterochromatin to the NL and repelling heterochromatin from nuclear pores. (*C*) Schematic of OIS induction in ER:Ras cells by 4-hydroxy-tamoxifen (4HT) and continued proliferation in ER:Stop cells. (*D*) Western blot showing POM121 (*left* panel) and TPR (*right* panel) levels in 4HT-treated ER:Stop and ER:Ras cells. (*E*) TPR immunostaining in ER:Stop and ER:Ras cells treated with 4HT. (*Left*) The bottom plane of a nucleus imaged by structured illuminated microscopy (SIM). (*Right*) Enlargement of the *insets*. Bars, 2 µm. (*F*) Mean (±SEM) nuclear pore density (pores per square micrometer) in 4HT-treated ER:Stop and ER:Ras cells as counted by TPR staining in three biological replicates. (***) *P* = 0.0001. (*G*) As in *F*, but for Pom121 staining. (h.s.) Highly significant (*P* = 1.3 × 10^−06^).

The composition and density of the NPC change during differentiation and tumorigenesis (D'Angelo et al. 2012; Raices and D'Angelo 2012; Sellés et al. 2017; Rodriguez-Bravo et al. 2018). We therefore hypothesized that the NPC could contribute to global chromatin organization and that, specifically, heterochromatin organization could result from a balance of forces attracting heterochromatin to the NL and forces repelling it away from the NPC (Fig. 1B). In support of this hypothesis, we show here that nuclear pore density increases during OIS and that this increase is necessary for heterochromatin reorganization into SAHFs. We identified TPR as a key player in this reorganization. Furthermore, we demonstrated the functional consequences of heterochromatin reorganization in OIS for the programmed activation of inflammatory cytokine gene expression: the senescence-associated secretory phenotype (SASP).

## Results and Discussion

### Nuclear pore density increases during OIS

To assess the role of the NPC in SAHF formation during OIS, we induced the activity of oncogenic Ras (RAS^G12V^) by addition of 4-hydroxy-tamoxifen (4HT) in human IMR90 cells, leading to OIS, activation of p53 and p16, and expression of SASP proteins (Fig. 1C; Supplemental Fig. S1A; Acosta et al. 2013). Nuclear pores disassemble upon entry into mitosis but are very stable during interphase (Daigle et al. 2001; Dultz and Ellenberg 2010). In quiescent cells, nuclear pore density is stabilized by down-regulation of nucleoporin mRNAs (D'Angelo et al. 2009). However, expression profiling in OIS cells (ER:Ras) showed that, compared with control ER:Stop (Stop codon) cells, nucleoporin mRNA levels are unchanged during senescence (Supplemental Fig. S1B). Nucleoporin protein accumulation in senescent cells was confirmed by immunoblotting for POM121 (an integral membrane protein of the NPC central ring) (Funakoshi et al. 2011) and TPR (a large coiled-coil protein of the nuclear basket) (Fig. 1A,D; Cordes et al. 1998). Immunofluorescence and structured illuminated microscopy (SIM) (Schermelleh et al. 2008) showed that increased nucleoporin levels during OIS results in an increased nuclear pore density (Fig. 1E–G).

### Decreasing nuclear pore density leads to loss of SAHF formation

**Figure 2.**
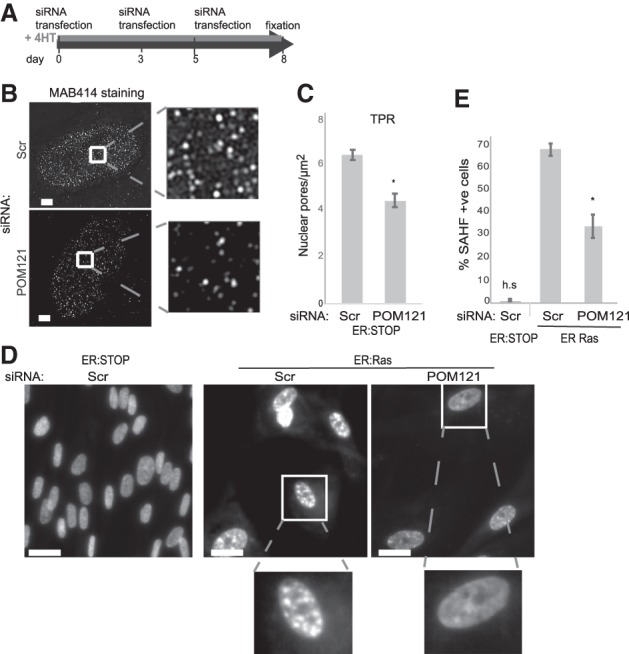
Increased nuclear pore density in OIS is necessary for SAHF formation. (*A*) Schematic showing the depletion experiment for *B*–*E*. (*B*) MAB414 (antibody recognizing several nucleoporins) immunostaining in ER:Stop cells treated with 4HT after 2 d of knockdown with scramble (Scr) or POM121 siRNAs. (*Left*) The bottom plane of the nucleus imaged by SIM. (*Right*) Enlargement of the *insets*. Bars, 2 µm. (*C*) Mean (±SEM) nuclear pore density (pores per square micrometer) in 4HT-treated ER:Stop cells after scramble (Scr) or POM121 siRNA knockdown, as assayed by TPR staining in three biological replicates, (*) *P* < 0.05. (*D*) DAPI staining of 4HT-treated ER:Stop and ER:Ras cells in controls (Scr) and upon POM121 depletion (siPOM121). Bars, 10 µm. (*Bottom*) Enlargement of the *insets*. (*E*) Mean (±SEM) percentage of cells containing SAHFs in 4HT-treated ER:Stop and ER:Ras cells after knockdown with scramble (Scr) siRNAs and in 4HT-treated ER:Ras cells with POM121 siRNAs. Data are from three experiments. (*) *P* < 0.05; (h.s.) highly significant.

To assess whether the increased nuclear pore density is responsible for heterochromatin reorganization into SAHFs, we used siRNAs to deplete POM121 (Supplemental Fig. S2A) during the entire course of OIS induction (Fig. 2A). As expected, since POM121 is required for NPC assembly during interphase (Dultz and Ellenberg 2010; Funakoshi et al. 2011), this led to a decrease in nuclear pore density (Fig. 2B,C; Supplemental Fig. S2B). Consistent with our hypothesis, POM121 depletion resulted in a reduction of OIS cells containing SAHFs (Fig. 2D,E).

### The nucleoporin TPR is necessary for SAHF formation and maintenance

TPR is the last nucleoporin to be incorporated in new NPCs (Bodoor et al. 1999) through its interaction with NUP153 (Fig. 1A; Hase and Cordes 2003). TPR has been shown to establish heterochromatin exclusion zones at nuclear pores (Krull et al. 2010) and influence HIV integration sites by maintaining an open chromatin architecture near the NPC (Lelek et al. 2015).

**Figure 3.**
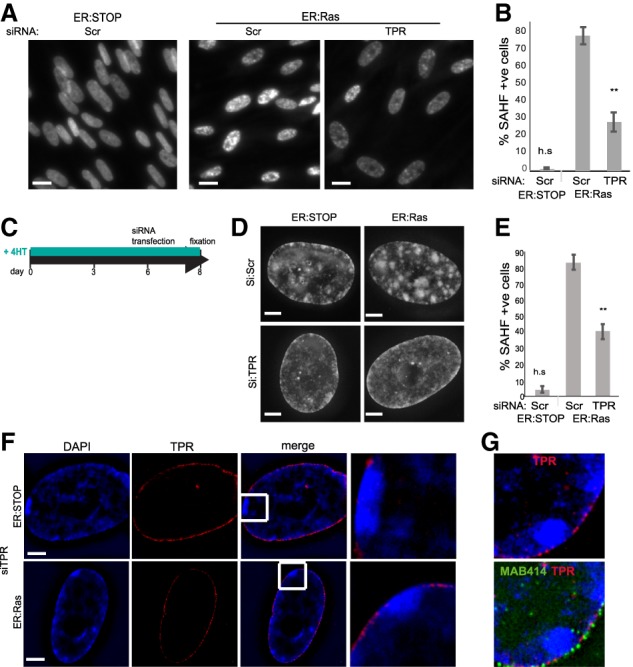
TPR is necessary for SAHF formation and maintenance. (*A*) DAPI staining of nonsenescent 4HT-treated ER:Stop and OIS (ER:Ras) cells after control scramble (Scr) siRNA and upon TPR depletion (siTPR). Bars, 10 µm. (*B*) Mean (±SEM) percentage of cells containing SAHFs in 4HT-treated ER:Stop and ER:Ras cells after knockdown siRNAs as in *A*. Data are from three experiments. (**) *P* < 0.01; (h.s.) highly significant. (*C*) Time course for TPR depletion by siRNA late in the OIS program as performed for *D*–*F*. (*D*) DAPI staining of 4HT-treated ER:Stop and OIS cells (ER:Ras) in controls (Scr) and upon TPR depletion (siTPR). Bars, 2 µm. (*E*) Mean (±SEM) percentage of cells containing SAHFs in 4HT-treated ER:Stop and ER:Ras cells after knockdown with scramble (Scr) siRNAs and in ER:Ras cells with TPR siRNAs. Data are from three experiments. (**) *P* < 0.01; (h.s.) highly significant. (*F*) DAPI (blue) and TPR (red) staining of 4HT-treated ER:Stop and ER:Ras upon TPR depletion (siTPR) imaged by SIM. (*Right*) Enlargement of the *insets*. Bars, 2 µm. (*G*, *top*) DAPI (blue) and TPR (red) staining of 4HT-treated ER:Ras cells upon TPR depletion. (*Bottom*) Costaining with the nucleoporin antibody MAB414 (green).

To determine whether it is the increased abundance of TPR at the nuclear periphery of OIS cells—as a result of elevated nuclear pore density—that is responsible for SAHF formation, we depleted TPR during OIS induction (Supplemental Fig. S3A,B). Contrary to a recent report, TPR depletion did not affect nuclear pore density (Supplemental Fig. S3C; McCloskey et al. 2018). However, similar to POM121 depletion, TPR depletion led to the loss of SAHFs (Fig. 3A,B). We confirmed these results with four independent siRNAs targeting TPR (Supplemental Fig. S3D–F). We conclude that TPR is necessary for the formation of SAHFs during OIS.

The effect of TPR knockdown on heterochromatin relocalization during OIS does not appear to be due to obvious changes in the amount of laminB1 at the NL (Supplemental Fig. S4A).

To assess whether TPR is necessary for maintenance as well as the formation of SAHFs, we used a time course to determine when SAHFs are formed. The percentage of cells containing SAHFs increased gradually after 4HT treatment of ER:Ras cells, reaching a maximum at 6 d (Supplemental Fig. S4B). We therefore depleted TPR 6 d after 4HT addition, when SAHFs have already formed (Fig. 3C). We observed a dramatic reduction of cells containing SAHFs 2 d later (day 8) (Fig. 3D,E). siRNA depletion under these conditions was only partial, and we observed loss of SAHFs in cells specifically depleted for TPR, whereas SAHFs were maintained in cells where knockdown was incomplete (Supplemental Fig. S4C). In some cells with partial TPR depletion, there was a relocalization of heterochromatin to the nuclear periphery in patches that corresponded to sites of TPR depletion (Fig. 3F) but that still contained nuclear pores as detected by MAB414 staining (Fig. 3G). We conclude that exclusion of heterochromatin from the nuclear periphery by TPR is necessary for both the formation and maintenance of SAHFs during OIS.

### TPR is necessary for the SASP

SAHFs are proposed to be involved in silencing promitotic genes, contributing to stable cell cycle arrest (Narita et al. 2003, 2006; Zhang et al. 2007). However, TPR-depleted OIS cells did not show defective cell cycle arrest as assayed by 5-bromo-2′-deoxyuridine (BrdU) incorporation and activation of p16, p21, and p53 (Supplemental Fig. S5A–C). This suggests that SAHFs are dispensable for cell cycle arrest, in agreement with the fact that not all senescent cells form SAHFs (Kosar et al. 2011). Furthermore, SAHFs have been shown to be insufficient to maintain cell cycle arrest, as inactivation of p53 or ATM in OIS cells leads to senescence escape without SAHF alteration (Di Micco et al. 2011).

**Figure 4.**
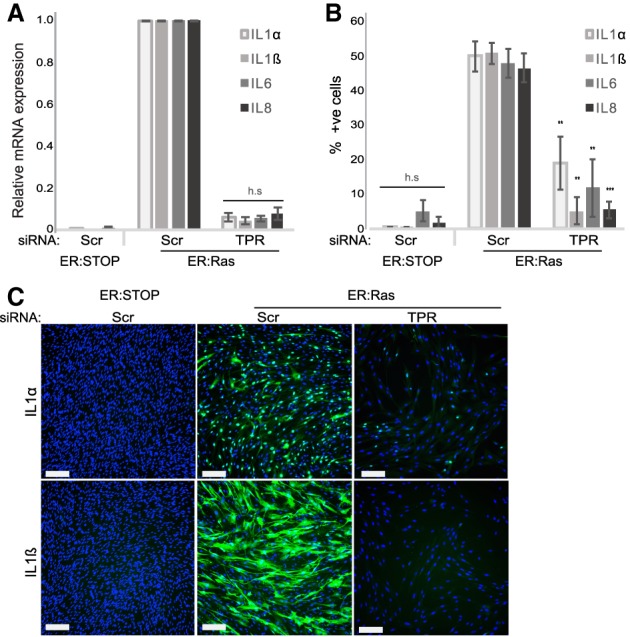
TPR is necessary for the SASP. (*A*) Mean (±SEM) mRNA level measured by quantitative RT–PCR (qRT–PCR) for SASP genes (*IL1A*, *IL1B*, *IL6*, and *IL8*) in 4HT-treated ER:Stop and ER:Ras cells after knockdown with scramble (Scr) siRNAs and in 4HT-treated ER:Ras cells with TPR siRNAs. Expression is relative to ER:Ras cells transfected with Scr siRNAs. Data are from three experiments. (h.s.) Highly significant. (*B*) Mean (±SEM) percentage of cells positive by immunostaining for SASP cytokines (IL1α, IL1β, IL6, and IL8) in 4HT-treated ER:Stop and ER:Ras cells after siRNA knockdown as in *A*. Data are from three experiments. (**) *P* < 0.01; (***) *P* < 0.001; (h.s.) highly significant. (*C*) Immunostaining (green) for IL1α and IL1β in DAPI-stained (blue) nuclei of 4HT-treated ER:Stop and ER:Ras cells subjected to RNAi as in *A*. Bars, 100 µm.

An important characteristic of OIS is activation of the SASP, which is responsible for the non-cell-autonomous effects of senescence. The SASP consists of the expression and secretion of cytokines, chemokines, extracellular matrix proteases, growth factors, and other signaling molecules. The SASP is a tumor-suppressive mechanism that reinforces cell cycle arrest and leads to paracrine senescence but can also promote tumor progression in premalignant lesions (Coppé et al. 2010; Acosta et al. 2013). Strikingly, in the absence of SAHFs after TPR depletion, we observed a complete loss of the SASP, as exemplified by a lack of IL1α, IL1β, IL6, and IL8 mRNA and protein (Fig. 4A–C; Supplemental Fig. S5D,E). SAHF and SASP loss upon TPR depletion does not seem to be due to a general defect in nuclear transport, as we detected NFκB nuclear import upon induction of paracrine senescence (Supplemental Fig. S6A–C; Acosta et al. 2008, 2013; Chien et al. 2011).

Similarly to some other nucleoporins, a fraction of TPR is present in the nucleoplasm as well as at nuclear pores (Frosst et al. 2002). To assess whether it is the increase in nuclear pore density in OIS (and consequent increased TPR abundance at the nuclear periphery) that is necessary for the SASP or whether TPR has an independent role, we assessed the SASP upon depletion of POM121, which is present only within the NPC. Decreased nuclear pore density upon POM121 depletion did not affect cell cycle arrest (Supplemental Fig. S7A), but the SASP was impaired (Supplemental Fig. S7B–D).

The nuclear pore basket nucleoporin NUP153 (Fig. 1A) is necessary for the association of TPR with the NPC (Hase and Cordes 2003). To further confirm that the role of TPR in SAHF formation and the SASP depends on its presence at the NPC rather than in the nucleoplasm, we depleted NUP153 (Supplemental Fig. S8A). NPC density was unchanged (Supplemental Fig. S8B), but, consistent with the role of NUP153 in TPR–nuclear basket association, TPR-containing NPC density decreased upon NUP153 depletion (Supplemental Fig. S8C). Concomitantly, the percentage of SAHF-containing cells decreased (Supplemental Fig. S8D,E), and the SASP was lost (Supplemental Fig. S8F). We conclude that it is TPR association with the NPC that is necessary for SAHF formation and SASP activation in OIS.

### Chromatin reorganization controls the SASP

**Figure 5.**
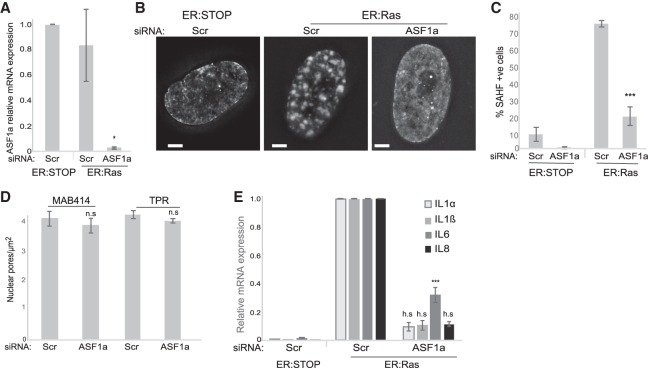
Chromatin reorganization seems necessary for the SASP. (*A*) Mean (±SEM) ASF1a mRNA level established by qRT–PCR in 4HT-treated ER:Stop and ER:Ras cells after knockdown with scramble (Scr) or ASF1a siRNAs. Expression is shown relative to ER:Stop cells transfected with Scr siRNAs. Data are from three experiments. (*) *P* < 0.05. (*B*) DAPI staining of 4HT-treated ER:Stop and ER:Ras cells in controls (Scr) and upon ASF1a depletion (siASF1a). Bars, 2 µm. (*C*) Mean (±SEM) percentage of cells containing SAHFs in 4HT-treated ER:Stop and ER:Ras cells after knockdown with scramble (Scr) siRNAs and in ER:Ras cells with ASF1a siRNAs. Data are from three experiments. (***) *P* < 0.001. (*D*) Mean (±SEM) nuclear pore density (pores per square micrometer) in 4HT-treated ER:Stop cells after knockdown with scramble (Scr) or ASF1a (siASF1a) siRNAs as counted by MAB414 or TPR staining in three biological replicates. (n.s.) Nonsignificant. (*E*) Mean (±SEM) mRNA levels measured by qRT–PCR for *IL1A*, *IL1B*, *IL6*, and *IL8* in 4HT-treated ER:Stop and ER:Ras cells after knockdown with scramble (Scr) siRNAs and in 4HT-treated ER:Ras cells with ASF1a siRNAs. Expression is shown relative to ER:Ras cells transfected with Scr siRNAs. Data are from three experiments. (***) *P* < 0.001; (h.s.) highly significant.

Our results suggest that heterochromatin reorganization is necessary for the SASP during OIS. To exclude that nuclear pores regulate the SASP through another independent mechanism, we used a different means to deplete SAHFs. The histone chaperone ASF1a is required for SAHF formation (Zhang et al. 2005, 2007), and, indeed, its depletion led to a loss of SAHFs in ER:Ras cells (Fig. 5A–C). ASF1a depletion did not affect nuclear pore density (Fig. 5D), but, as for TPR and POM121 depletion, there is a dramatic loss of the SASP upon ASF1a depletion in ER:Ras cells (Fig. 5E). While we cannot completely rule out that intact nuclear pores are needed for SASP activation independent of chromatin reorganization, this result supports the hypothesis that heterochromatin reorganization is necessary for the SASP.

Our data suggest that an increase in nuclear pore density is responsible for the eviction of heterochromatin from the nuclear periphery by TPR and the consequent formation of SAHFs in OIS. Similar mechanisms could be conserved in other types of senescence, as nuclear pore density is also increased in replicative senescence (Maeshima et al. 2006). Chromatin organization relative to the nuclear periphery has generally been considered from the point of view of interactions between (hetero)chromatin and components of the NL. Here we demonstrate that the repulsion of heterochromatin by nuclear pores is another important principle of nuclear organization, and it will be interesting to establish whether the modulation of nuclear pore density also influences the 3D organization of the genome during development.

## Materials and methods

### Cell culture

IMR90 cells were infected with pLNC-ER:RAS and pLXS-ER:Stop retroviral vectors to produce ER:Ras and ER:Stop cells, respectively (Acosta et al. 2013). Ras translocation to the nucleus was induced by addition of 4HT (Sigma) diluted in DMSO to 100 nM. 4HT-containing medium was changed every 3 d.

### siRNA transfection

IMR90, ER:Stop, and ER:Ras cells (2 × 10^5^) were transfected using Dharmafect transfection reagent (Dharmacon) with a 30 nM final concentration of predesigned siRNAs (Dharmacon) (Supplemental Table S1).

### RNA expression analysis

mRNA expression profiling was by IonTorrent mRNA sequencing using the Ion AmpliSeq transcriptome human gene expression kit. Six biological replicates were analyzed, and adjusted *P*-values were calculated by Benjamini and Hochberg (BH) and false discovery rate multiple test correction. Data analysis was performed using Babelomics-5 (http://babelomics.bioinfo.cipf.es).

For individual mRNAs, total RNA was extracted using the RNeasy minikit (Qiagen), and cDNAs were generated using SuperScript II (Life technologies). Real-time PCR was performed on a LightCycler 480 (Roche) using SYBR Green PCR master mix (Roche) using the primers listed in Supplemental Table S3. Expression was normalized to *β-actin*.

### Immunoblotting

Cells (1 × 10^6^) were lysed in RIPA buffer, and protein concentration was determined using a Pierce BCA protein analysis kit. Fifteen micrograms of proteins was run into NuPage 3%–8% Tris acetate gels (Invitrogen). After transfer onto nitrocellulose with a iBlot 2 gel transfer device (Thermo Fisher), immunoblotting was done using antibodies listed in Supplemental Table S2.

### Immunofluorescence and SAHF measurement

Cells (2 × 10^5^) were seeded and grown on coverslips during senescence induction. Cells were fixed in 4% paraformaldehyde (pFa) for 10 min at room temperature, permeabilized in 0.1% Triton X-100 for 10 min, blocked in 1% BSA for 30 min, and incubated with primary antibodies diluted in 1% BSA for 1 h and with fluorescently labeled secondary antibodies (Life Technologies) for 45 min. Coverslips were counterstained with DAPI and mounted in VectaShield (Vector Laboratories).

To detect replicating cells, cells were incubated with 10 μM BrdU (Sigma) for 16 h prior to fixation and immunodetection using a BrdU antibody (BD Pharmingene, 555627) in the presence of 1 mM MgCl_2_ and 0.5 U/µL DNaseI (Sigma, D4527).

Detection of SASP proteins, tumor suppressors, and BrdU-positive cells by high-content microscopy is described in Hari and Acosta (2017). The percentage of SAHF-positive cells was determined by manual examination of 100–200 DAPI-stained cells.

### SIM and measurement of nuclear pore density

The bottom plane of cells was imaged by 3D SIM (Nikon N-SIM) and reconstructed using NIS element software after immunofluorescence with antibodies as indicated in Supplemental Table S2. Fifteen nuclei were imaged for each condition, and five regions of interest (ROIs) of 100 × 100 pixels were analyzed per nucleus. Individual NPCs in each ROI were counted manually.

### β-Galactosidase staining

Senescence-associated β-galactosidase staining solution was prepared using 20× KC [100 mM K_3_Fe (CN)_6_, 100 mM K_4_Fe (CN)_6_*3H_2_O in PBS] and 20× X-Gal solution (Thermo Fisher Scientific) diluted to 1× in PBS/1 mM MgCl_2_ (pH 5.5–6). Staining was conducted overnight on glutaraldehyde-fixed cells.

### Statistics

All experiments were performed in a minimum of three biological replicates. Error bars are standard error of the mean. *P*-values were obtained by two-sample equal variance two-tailed *t*-test.

## Supplementary Material

Supplemental Material

## References

[GAD321117BOUC1] Acosta JC, O'Loghlen A, Banito A, Guijarro MV, Augert A, Raguz S, Fumagalli M, Da Costa M, Brown C, Popov N, 2008 Chemokine signaling via the CXCR2 receptor reinforces senescence. Cell 133: 1006–1018. 10.1016/j.cell.2008.03.038 18555777

[GAD321117BOUC2] Acosta JC, Banito A, Wuestefeld T, Georgilis A, Janich P, Morton JP, Athineos D, Kang T-W, Lasitschka F, Andrulis M, 2013 A complex secretory program orchestrated by the inflammasome controls paracrine senescence. Nat Cell Biol 15: 978–990. 10.1038/ncb2784 23770676 PMC3732483

[GAD321117BOUC3] Bodoor K, Shaikh S, Salina D, Raharjo WH, Bastos R, Lohka M, Burke B. 1999 Sequential recruitment of NPC proteins to the nuclear periphery at the end of mitosis. J Cell Sci 112: 2253–2264. 10362555 10.1242/jcs.112.13.2253

[GAD321117BOUC4] Capelson M, Hetzer MW. 2009 The role of nuclear pores in gene regulation, development and disease. EMBO Rep 10: 697–705. 10.1038/embor.2009.147 19543230 PMC2727434

[GAD321117BOUC5] Chandra T, Kirschner K, Thuret J-Y, Pope BD, Ryba T, Newman S, Ahmed K, Samarajiwa SA, Salama R, Carroll T, 2012 Independence of repressive histone marks and chromatin compaction during senescent heterochromatic layer formation. Mol Cell 47: 203–214. 10.1016/j.molcel.2012.06.010 22795131 PMC3701408

[GAD321117BOUC6] Chien Y, Scuoppo C, Wang X, Fang X, Balgley B, Bolden JE, Premsrirut P, Luo W, Chicas A, Lee CS, 2011 Control of the senescence-associated secretory phenotype by NF-κB promotes senescence and enhances chemosensitivity. Genes Dev 25: 2125–2136. 10.1101/gad.17276711 21979375 PMC3205583

[GAD321117BOUC7] Coppé J-P, Desprez P-Y, Krtolica A, Campisi J. 2010 The senescence-associated secretory phenotype: the dark side of tumor suppression. Annu Rev Pathol 5: 99–118. 10.1146/annurev-pathol-121808-102144 20078217 PMC4166495

[GAD321117BOUC8] Cordes VC, Hase ME, Müller L. 1998 Molecular segments of protein Tpr that confer nuclear targeting and association with the nuclear pore complex. Exp Cell Res 245: 43–56. 10.1006/excr.1998.4246 9828100

[GAD321117BOUC9] Daigle N, Beaudouin J, Hartnell L, Imreh G, Hallberg E, Lippincott-Schwartz J, Ellenberg J. 2001 Nuclear pore complexes form immobile networks and have a very low turnover in live mammalian cells. J Cell Biol 154: 71–84. 10.1083/jcb.200101089 11448991 PMC2196857

[GAD321117BOUC10] D'Angelo MA, Raices M, Panowski SH, Hetzer MW. 2009 Age-dependent deterioration of nuclear pore complexes causes a loss of nuclear integrity in postmitotic cells. Cell 136: 284–295. 10.1016/j.cell.2008.11.037 19167330 PMC2805151

[GAD321117BOUC11] D'Angelo MA, Gomez-Cavazos JS, Mei A, Lackner DH, Hetzer MW. 2012 A change in nuclear pore complex composition regulates cell differentiation. Dev Cell 22: 446–458. 10.1016/j.devcel.2011.11.021 22264802 PMC3288503

[GAD321117BOUC12] Di Micco R, Sulli G, Dobreva M, Liontos M, Botrugno OA, Gargiulo G, dal Zuffo R, Matti V, d'Ario G, Montani E, 2011 Interplay between oncogene-induced DNA damage response and heterochromatin in senescence and cancer. Nat Cell Biol 13: 292–302. 10.1038/ncb2170 21336312 PMC3918344

[GAD321117BOUC14] Dultz E, Ellenberg J. 2010 Live imaging of single nuclear pores reveals unique assembly kinetics and mechanism in interphase. J Cell Biol 191: 15–22. 10.1083/jcb.201007076 20876277 PMC2953446

[GAD321117BOUC15] Frosst P, Guan T, Subauste C, Hahn K, Gerace L. 2002 Tpr is localized within the nuclear basket of the pore complex and has a role in nuclear protein export. J Cell Biol 156: 617–630. 10.1083/jcb.200106046 11839768 PMC2174070

[GAD321117BOUC16] Funakoshi T, Clever M, Watanabe A, Imamoto N. 2011 Localization of Pom121 to the inner nuclear membrane is required for an early step of interphase nuclear pore complex assembly. Mol Biol Cell 22: 1058–1069. 10.1091/mbc.e10-07-0641 21289085 PMC3069009

[GAD321117BOUC17] Garcia-Segura LM, Lafarga M, Berciano MT, Hernandez P, Andres MA. 1989 Distribution of nuclear pores and chromatin organization in neurons and glial cells of the rat cerebellar cortex. J Comp Neurol 290: 440–450. 10.1002/cne.902900311 2592622

[GAD321117BOUC18] Guelen L, Pagie L, Brasset E, Meuleman W, Faza MB, Talhout W, Eussen BH, de Klein A, Wessels L, de Laat W, 2008 Domain organization of human chromosomes revealed by mapping of nuclear lamina interactions. Nature 453: 948–951. 10.1038/nature06947 18463634

[GAD321117BOUC19] Hari P, Acosta JC. 2017 Detecting the senescence-associated secretory phenotype (SASP) by high content microscopy analysis. In Oncogene-induced senescence: methods and protocols (ed. Nikiforov MA), pp. 99–109. Springer, New York. 10.1007/978-1-4939-6670-7_9 27812871

[GAD321117BOUC20] Hase ME, Cordes VC. 2003 Direct interaction with Nup153 mediates binding of Tpr to the periphery of the nuclear pore complex. Mol Biol Cell 14: 1923–1940. 10.1091/mbc.e02-09-0620 12802065 PMC165087

[GAD321117BOUC22] Kim SJ, Fernandez-Martinez J, Nudelman I, Shi Y, Zhang W, Raveh B, Herricks T, Slaughter BD, Hogan JA, Upla P, 2018 Integrative structure and functional anatomy of a nuclear pore complex. Nature 555: 475–482. 10.1038/nature26003 29539637 PMC6022767

[GAD321117BOUC23] Kosar M, Bartkova J, Hubackova S, Hodny Z, Lukas J, Bartek J. 2011 Senescence-associated heterochromatin foci are dispensable for cellular senescence, occur in a cell type- and insult-dependent manner and follow expression of p16ink4a. Cell Cycle 10: 457–468. 10.4161/cc.10.3.14707 21248468

[GAD321117BOUC24] Krull S, Dörries J, Boysen B, Reidenbach S, Magnius L, Norder H, Thyberg J, Cordes VC. 2010 Protein Tpr is required for establishing nuclear pore-associated zones of heterochromatin exclusion. EMBO J 29: 1659–1673. 10.1038/emboj.2010.54 20407419 PMC2876962

[GAD321117BOUC25] Lelek M, Casartelli N, Pellin D, Rizzi E, Souque P, Severgnini M, Di Serio C, Fricke T, Diaz-Griffero F, Zimmer C, 2015 Chromatin organization at the nuclear pore favours HIV replication. Nat Commun 6: 6483 10.1038/ncomms7483 25744187 PMC4366494

[GAD321117BOUC26] Lenain C, de Graaf CA, Pagie L, Visser NL, de Haas M, de Vries SS, Peric-Hupkes D, van Steensel B, Peeper DS. 2017 Massive reshaping of genome–nuclear lamina interactions during oncogene-induced senescence. Genome Res 27: 1634–1644. 10.1101/gr.225763.117 28916540 PMC5630027

[GAD321117BOUC27] Lukášová E, Kovařík A, Bačíková A, Falk M, Kozubek S. 2017 Loss of lamin B receptor is necessary to induce cellular senescence. Biochem J 474: 281–300. 10.1042/BCJ20160459 27760841

[GAD321117BOUC28] Maeshima K, Yahata K, Sasaki Y, Nakatomi R, Tachibana T, Hashikawa T, Imamoto F, Imamoto N. 2006 Cell-cycle-dependent dynamics of nuclear pores: pore-free islands and lamins. J Cell Sci 119: 4442–4451. 10.1242/jcs.03207 17074834

[GAD321117BOUC29] McCloskey A, Ibarra A, Hetzer MW. 2018 Tpr regulates the total number of nuclear pore complexes per cell nucleus. Genes Dev 32: 1321–1331. 10.1101/gad.315523.118 30228202 PMC6169833

[GAD321117BOUC30] Narita M, Nuñez S, Heard E, Narita M, Lin AW, Hearn SA, Spector DL, Hannon GJ, Lowe SW. 2003 Rb-mediated heterochromatin formation and silencing of E2F target genes during cellular senescence. Cell 113: 703–716. 10.1016/S0092-8674(03)00401-X 12809602

[GAD321117BOUC31] Narita M, Narita M, Krizhanovsky V, Nuñez S, Chicas A, Hearn SA, Myers MP, Lowe SW. 2006 A novel role for high-mobility group A proteins in cellular senescence and heterochromatin formation. Cell 126: 503–514. 10.1016/j.cell.2006.05.052 16901784

[GAD321117BOUC32] Raices M, D'Angelo MA. 2012 Nuclear pore complex composition: a new regulator of tissue-specific and developmental functions. Nat Rev Mol Cell Biol 13: 687–699. 10.1038/nrm3461 23090414

[GAD321117BOUC33] Rodriguez-Bravo V, Pippa R, Song W-M, Carceles-Cordon M, Dominguez-Andres A, Fujiwara N, Woo J, Koh AP, Ertel A, Lokareddy RK, 2018 Nuclear pores promote lethal prostate cancer by increasing POM121-driven E2F1, MYC, and AR nuclear import. Cell 174: 1200–1215.e20. 10.1016/j.cell.2018.07.015 30100187 PMC6150493

[GAD321117BOUC34] Sadaie M, Salama R, Carroll T, Tomimatsu K, Chandra T, Young ARJ, Narita M, Pérez-Mancera PA, Bennett DC, Chong H, 2013 Redistribution of the Lamin B1 genomic binding profile affects rearrangement of heterochromatic domains and SAHF formation during senescence. Genes Dev 27: 1800–1808. 10.1101/gad.217281.113 23964094 PMC3759696

[GAD321117BOUC35] Schermelleh L, Carlton PM, Haase S, Shao L, Winoto L, Kner P, Burke B, Cardoso MC, Agard DA, Gustafsson MGL, 2008 Subdiffraction multicolor imaging of the nuclear periphery with 3D structured illumination microscopy. Science 320: 1332–1336. 10.1126/science.1156947 18535242 PMC2916659

[GAD321117BOUC36] Sellés J, Penrad-Mobayed M, Guillaume C, Fuger A, Auvray L, Faklaris O, Montel F. 2017 Nuclear pore complex plasticity during developmental process as revealed by super-resolution microscopy. Sci Rep 7: 14732 10.1038/s41598-017-15433-2 29116248 PMC5677124

[GAD321117BOUC37] Solovei I, Wang AS, Thanisch K, Schmidt CS, Krebs S, Zwerger M, Cohen TV, Devys D, Foisner R, Peichl L, 2013 LBR and lamin A/C sequentially tether peripheral heterochromatin and inversely regulate differentiation. Cell 152: 584–598. 10.1016/j.cell.2013.01.009 23374351

[GAD321117BOUC38] Zhang R, Poustovoitov MV, Ye X, Santos HA, Chen W, Daganzo SM, Erzberger JP, Serebriiskii IG, Canutescu AA, Dunbrack RL, 2005 Formation of MacroH2A-containing senescence-associated heterochromatin foci and senescence driven by ASF1a and HIRA. Dev Cell 8: 19–30. 10.1016/j.devcel.2004.10.019 15621527

[GAD321117BOUC39] Zhang R, Chen W, Adams PD. 2007 Molecular dissection of formation of senescence-associated heterochromatin foci. Mol Cell Biol 27: 2343–2358. 10.1128/MCB.02019-06 17242207 PMC1820509

