# Supplementary material for: Nuclear pore density controls heterochromatin reorganization during senescence
Source: Genes Dev. 2019 Feb 1;33(3-4):144–9. doi: 10.1101/gad.321117.118 (PMC6362808; doi:10.1101/gad.321117.118)
Supplement: Supplemental Material [file supp_33_3-4_144__index.html]

Nuclear pore density controls heterochromatin reorganization during senescence — Supplemental Material 

# Nuclear pore density controls heterochromatin reorganization during senescence

## Supplemental Material

- Supplementary\_Data.pdf
