# Supplementary material for: Nuclear pore density controls heterochromatin reorganization during senescence
Source: Genes Dev. 2019 Feb 1;33(3-4):144–9. doi: 10.1101/gad.321117.118 (PMC6362808; doi:10.1101/gad.321117.118)
Supplement: Supplemental Material [file supp_gad.321117.118_Supplementary_Data.pdf]

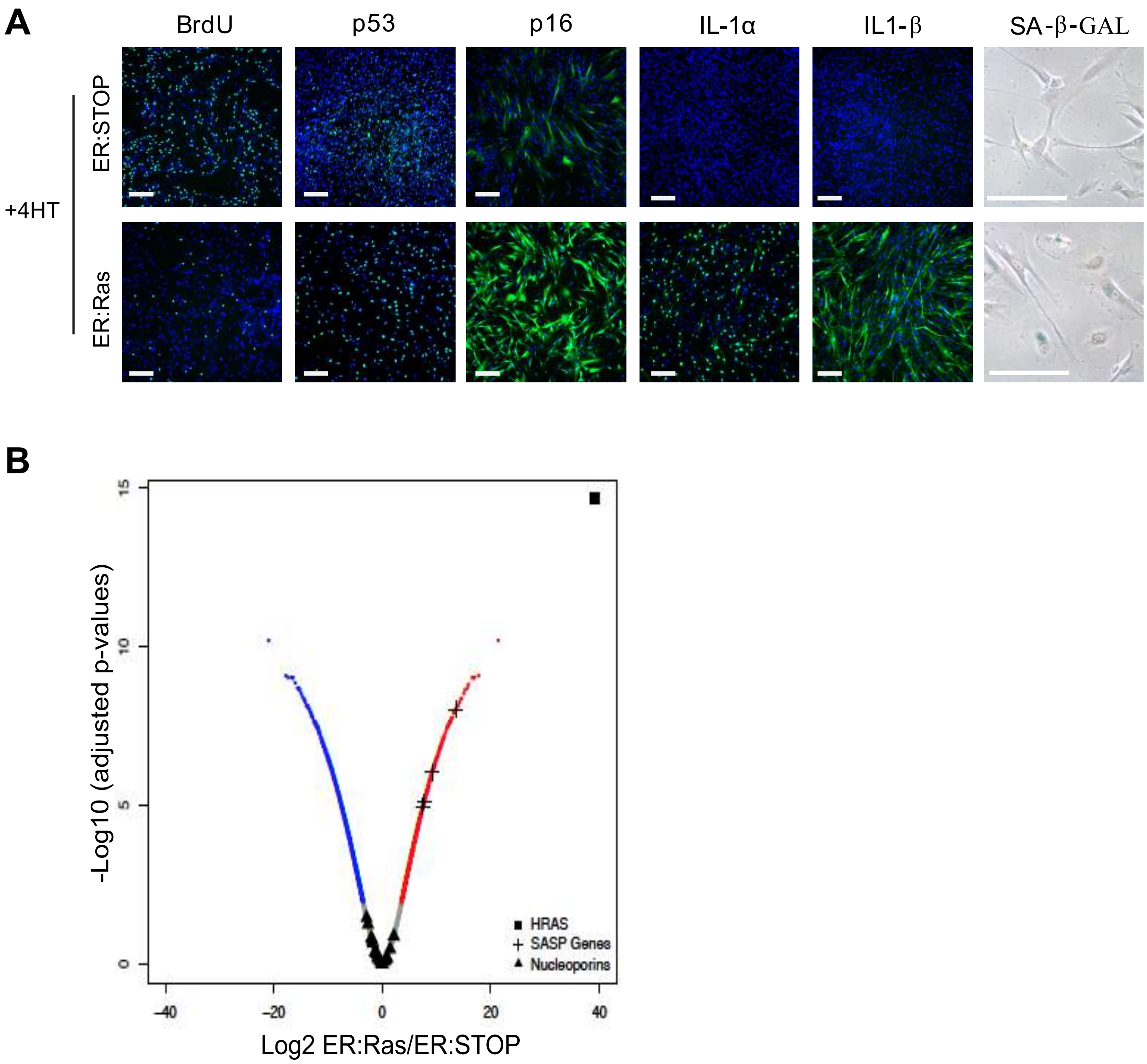

Supplementary Figure 1- OIS induction in ER:Ras cells

A) Immunostaining (green) for BrdU, p53, p16, IL1α, IL1β in DAPI (blue) stained nuclei of 4HT-treated ER:STOP (top row) and ER:Ras (bottom row) cells. Right hand panels shows senescence associated β-Galactosidase (SA-β-GAL) staining. Scale bars 100μm.

B) Volcano plot of genome wide mRNA expression analysis. Genes significantly down- or up- regulated in 4HT treated ER:Ras cells in comparision with 4HT treated ER:STOP are highlighted in blue and red respectively. Square shows the enrichment of Ras mRNA in ER:Ras cells, crosses show the upregulation of mRNAs for SASP genes, and nucleoporins are shown as triangles.

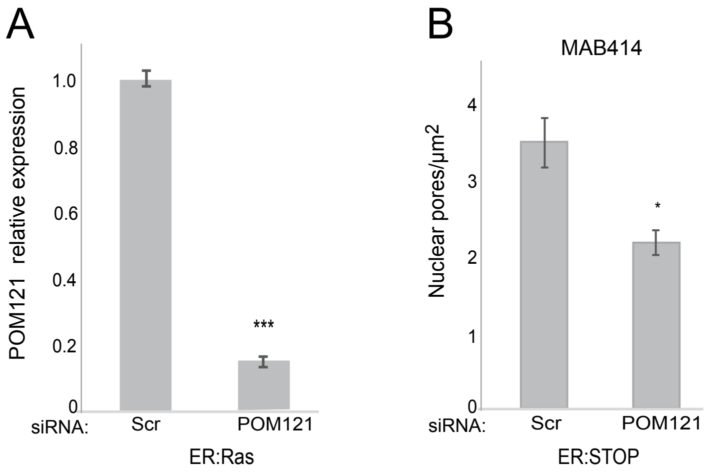

Supplementary Figure 2 - POM121 depletion leads to decreased nuclear pores density

A) Mean (± SEM) mRNA level, established by qRT-PCR, for POM121, in 4HT-treated ER:Ras cells after knockdown with scramble (Scr) or POM121 siRNAs. Expression is shown relative to ER:Ras cells transfected with Scr siRNAs. Data from 3 independent experiments. ***$p<0.001$

B) Mean (+/- SEM) nuclear pore density (pores/μm^2) in 4HT treated ER:Stop cells after knock down with scramble (SCR) or POM121 siRNAs as assayed by MAB414 staining in 3 independent biological replicates, *$p<0.05$,

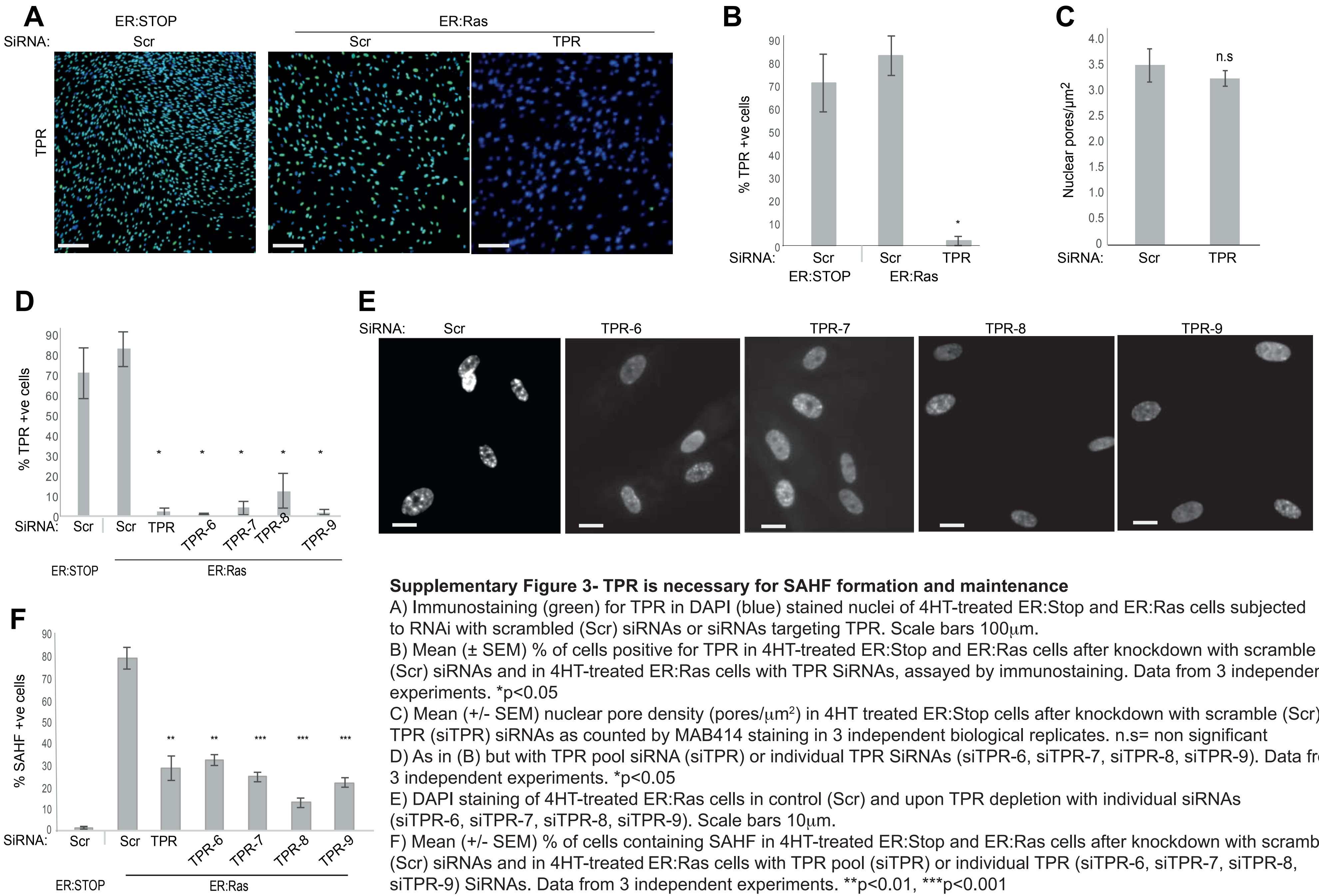

Supplementary Figure 3- TPR is necessary for SAHF formation and maintenance

A) Immunostaining (green) for TPR in DAPI (blue) stained nuclei of 4HT-treated ER:Stop and ER:Ras cells subjected to RNAi with scrambled (Scr) siRNAs or siRNAs targeting TPR. Scale bars 100μm.

B) Mean (± SEM) % of cells positive for TPR in 4HT-treated ER:Stop and ER:Ras cells after knockdown with scramble (Scr) siRNAs and in 4HT-treated ER:Ras cells with TPR SiRNAs, assayed by immunostaining. Data from 3 independent experiments. *p<0.05

C) Mean (+/- SEM) nuclear pore density (pores/μm^2) in 4HT treated ER:Stop cells after knockdown with scramble (Scr) or TPR (siTPR) siRNAs as counted by MAB414 staining in 3 independent biological replicates. n.s= non significant

D) As in (B) but with TPR pool siRNA (siTPR) or individual TPR SiRNAs (siTPR-6, siTPR-7, siTPR-8, siTPR-9). Data from 3 independent experiments. *p<0.05

E) DAPI staining of 4HT-treated ER:Ras cells in control (Scr) and upon TPR depletion with individual siRNAs (siTPR-6, siTPR-7, siTPR-8, siTPR-9). Scale bars 10μm.

F) Mean (+/- SEM) % of cells containing SAHF in 4HT-treated ER:Stop and ER:Ras cells after knockdown with scramble (Scr) siRNAs and in 4HT-treated ER:Ras cells with TPR pool (siTPR) or individual TPR (siTPR-6, siTPR-7, siTPR-8, siTPR-9) SiRNAs. Data from 3 independent experiments. **p<0.01, ***p<0.001

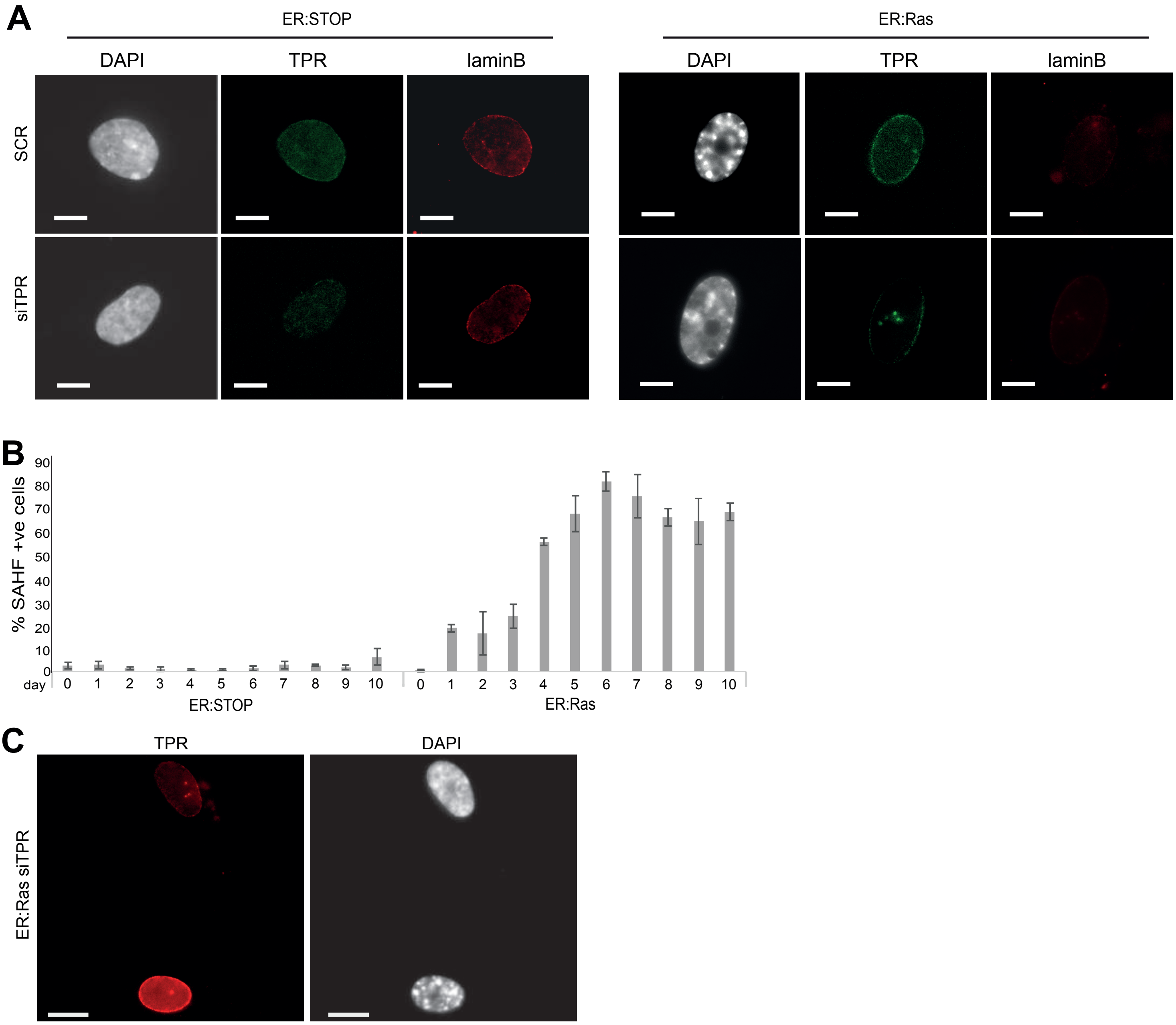

Supplementary Figure 4 - Analysis of Lamin B1 and SAHFs after TPR knockdown

A) Immunostaining for TPR (green), lamin B1 (red) and DAPI (white) stained nuclei of 4HT-treated ER:Stop and ER:Ras cells subjected to RNAi with siRNAs targeting TPR or with a scramled siRNA control (Scr). Scale bars 10μm

B) Time course of mean (+/- SEM) % cells with SAHF after 4HT-treatment of control (ER:Stop) and OIS cells (ER:Ras). Data from 3 experiments.

C) Immunostaining (red) for TPR and DAPI (white) stained nuclei of 4HT-treated ER:Stop and ER:Ras cells subjected to RNAi with siRNAs targeting TPR. SAHF positive cells correlate with TPR depleted cells. Scale bars 10μm.

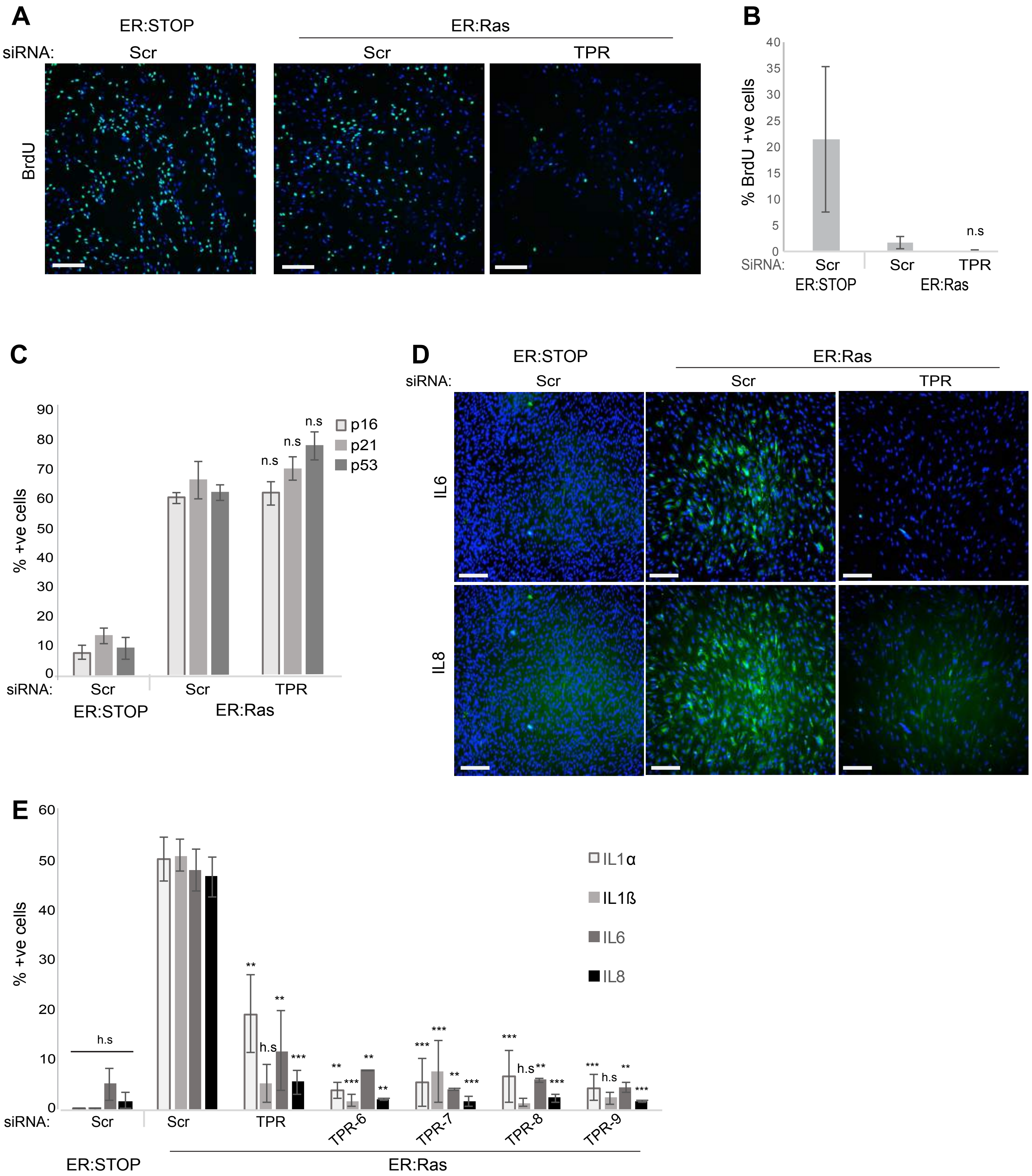

A
ER:STOP
ER:Ras
siRNA:
Scr
Scr
TPR
BrdU
B
% BrdU +ve cells
40
35
30
25
20
15
10
5
0
n.s
SiRNA:
Scr
Scr
TPR
ER:STOP
ER:Ras
C
% +ve cells
90
80
70
60
50
40
30
20
10
0
n.s
n.s
n.s
p16
p21
p53
siRNA:
Scr
Scr
TPR
ER:STOP
ER:Ras
D
ER:STOP
ER:Ras
siRNA:
Scr
Scr
TPR
IL6
IL8
E
% +ve cells
60
50
40
30
20
10
0
h.s
IL1α
IL1ß
IL6
IL8
siRNA:
Scr
Scr
TPR
TPR-6
TPR-7
TPR-8
TPR-9
ER:STOP
ER:Ras

Fig. S5

Supplementary Figure 5 Nuclear pores density regulates SASP

A) Immunostaining for BrdU (green) in 4HT-treated ER:Stop and ER:Ras cells after knockdown with scramble (Scr) siRNAs and in 4HT-treated ER:Ras cells with TPR. Nuclei are counterstained with DAPI (blue).

B) Mean (± SEM) % of cells positive for BrdU in 4HT-treated ER:Stop and ER:Ras cells after knockdown with scramble (Scr) siRNAs and in 4HT-treated ER:Ras cells with TPR siRNAs, measured as in (A). Data from 3 independent experiments. n.s = non significant.

C Mean (± SEM) % of cells positive for p16, p21 and p53 in 4HT-treated ER:Stop and ER:Ras cells after knockdown with scramble (Scr) siRNAs and in 4HT-treated ER:Ras cells with TPR siRNAs. Data from 3 independent experiments. n.s = non significant.

D) Immunostaining (green) for SASP cytokines IL6 and IL8 in DAPI (blue) stained nuclei of 4HT-treated ER:Stop and ER:Ras cells subjected to RNAi with scrambled (Scr) siRNAs or siRNAs targeting TPR. Scale bars 100μm.

E) Mean (± SEM) % of cells positive for SASP cytokines (IL1α, IL1β, IL6, IL8) in 4HT-treated ER:Stop and ER:Ras cells after knockdown with scramble (Scr) siRNAs and in 4HT-treated ER:Ras cells with a TPR siRNA pool and with individual TPR siRNAs, assayed by immunostaining. Data from 3 independent experiments. *p<0.05, **p<0.01, h.s=highly significant.

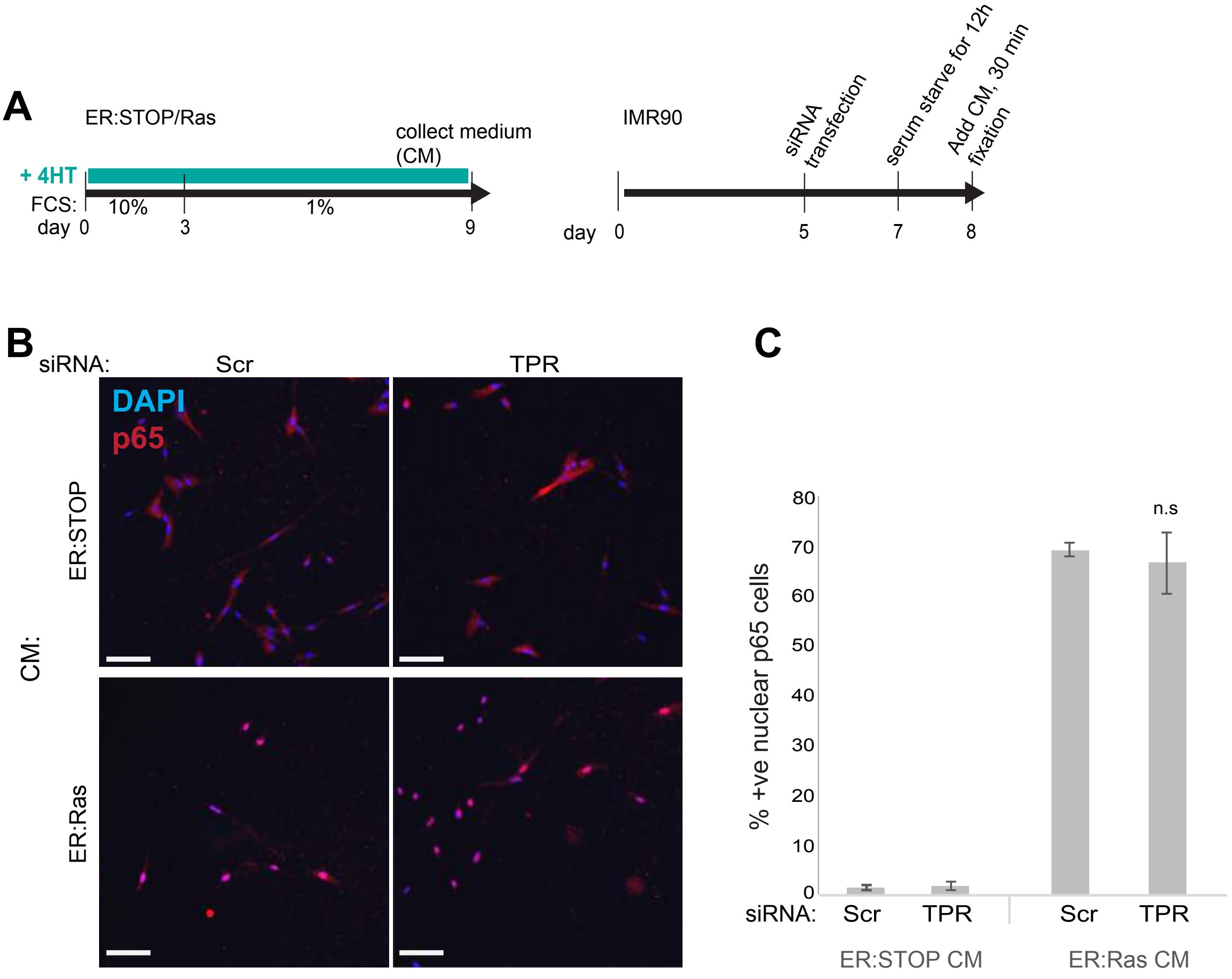

Figure S6. Nuclear import after TPR depletion

A) Schematic of NFκb nuclear import assessment as performed for panels B and C. Conditioned medium (CM) of ER:STOP and Ras cells was collected after 9 days of culture in presence of 4HT. CM was then added to IMR90 cells upon scramble (Scr) or TPR depletion by siRNA. NFκb-p65 nuclear import in IMR90 cells was assessed 30min after CM addition.
B) Immunostaining (red) for p65 in DAPI (blue) stained cells for scramble (Scr) or TPR (siTPR) depleted IMR90 cells upon addition of CM from 4HT-treated ER:Stop and ER:Ras cells. Scale bars 100μm.
C) Mean (+/- SEM) % of cells with nuclear p65 staining in scramble (Scr) or TPR (siTPR) depleted IMR90 cells upon addition of CM from 4HT-treated ER:Stop and ER:Ras cells. Data from 3 independent experiments. n.s=non significant.

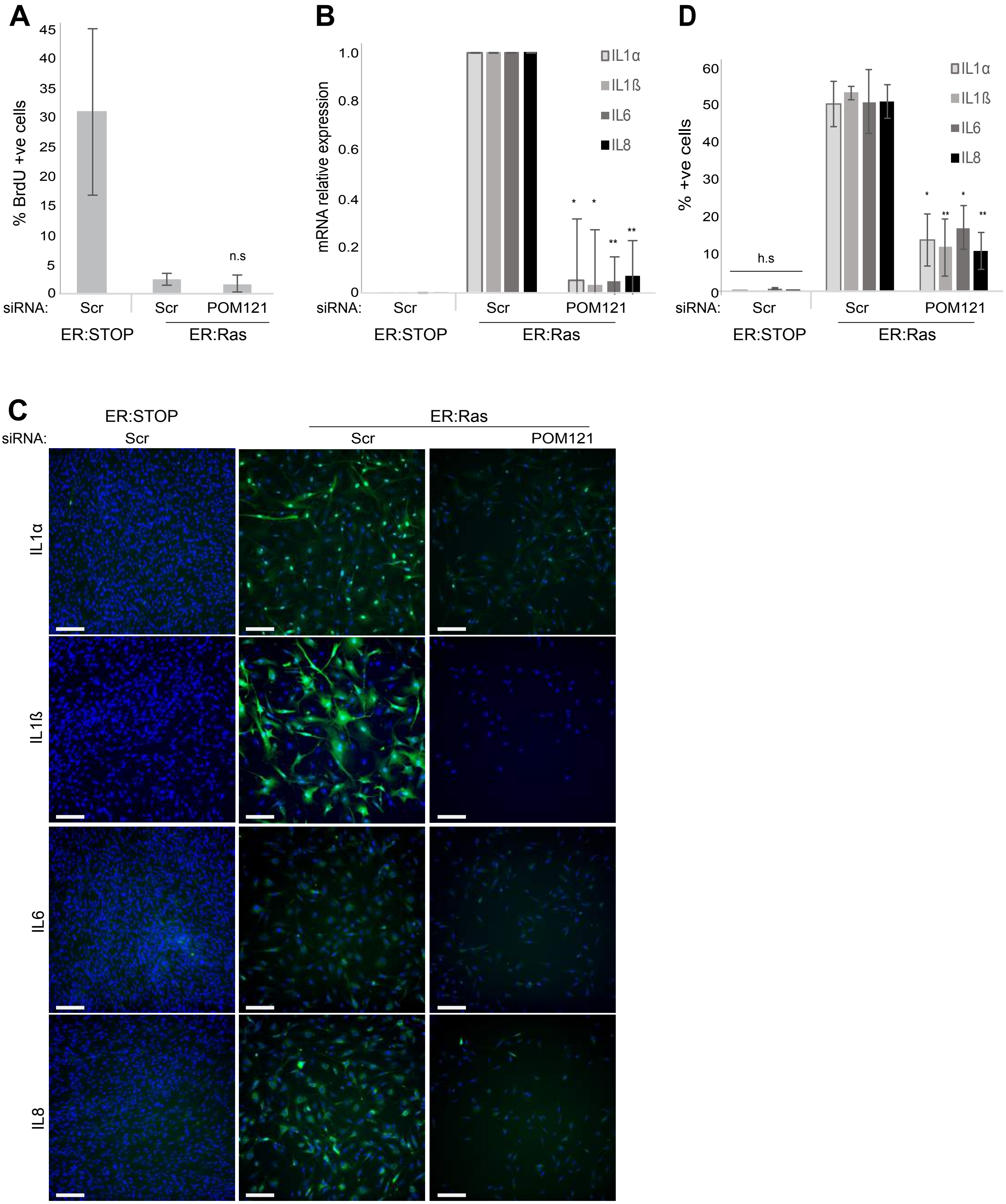
A
% BrdU +ve cells
45
40
35
30
25
20
15
10
5
0
n.s
siRNA:
Scr
Scr
POM121
ER:STOP
ER:Ras
B
mRNA relative expression
1.0
0.8
0.6
0.4
0.2
0
IL1α
IL1ß
IL6
IL8
*
*
**
**
siRNA:
Scr
Scr
POM121
ER:STOP
ER:Ras
D
% +ve cells
60
50
40
30
20
10
0
IL1α
IL1ß
IL6
IL8
h.s
*
**
*
**
siRNA:
Scr
Scr
POM121
ER:STOP
ER:Ras
C
ER:STOP
ER:Ras
siRNA:
Scr
Scr
POM121
IL1α
IL1ß
IL6
IL8

Fig. S7

Supplementary Figure 7 - nuclear pores density regulates SASP

A) Mean (± SEM) % of cells positive for BrdU in 4HT-treated ER:Stop and ER:Ras cells after knockdown with scramble (SCR) siRNAs and in 4HT-treated ER:Ras cells with Pom121 (siPOM121) siRNAs, measured upon immunostaining. Data from 3 independent experiments. n.s=non significant

B) Mean (± SEM) mRNA level, established by quantitative reverse transcription polymerase chain reaction (RT-PCR), for SASP genes (IL1α, IL1β, IL6, IL8), in 4HT-treated ER:Stop and ER:Ras cells after knockdown with scramble (Scr) siRNAs and in 4HT-treated ER Ras cells with Pom121 siRNAs. Expression is shown relative to ER Ras cells transfected with Scr siRNAs. Data from 3 independent experiments. $^{*}p<0.05$, $^{**}p<0.01$

C) Immunostaining (green) for SASP cytokines IL1α, IL1β, IL6 and IL8 in DAPI (blue) stained nuclei of 4HT-treated ER:Stop and ER:Ras cells subjected to RNAi with scrambled (Scr) siRNAs or siRNAs targeting POM121. Scale bars 100μm.

D) Mean (± SEM) % of cells positive for SASP cytokines (IL1α, IL1β, IL6, IL8) in 4HT-treated ER:Stop and ER:Ras cells after knockdown with scramble (Scr) siRNAs and in 4HT-treated ER:Ras cells with POM121 siRNAs, assayed by immunostaining. Data from 3 independent experiments. $^{*}p<0.05$, $^{**}p<0.01$, h.s=highly significant.

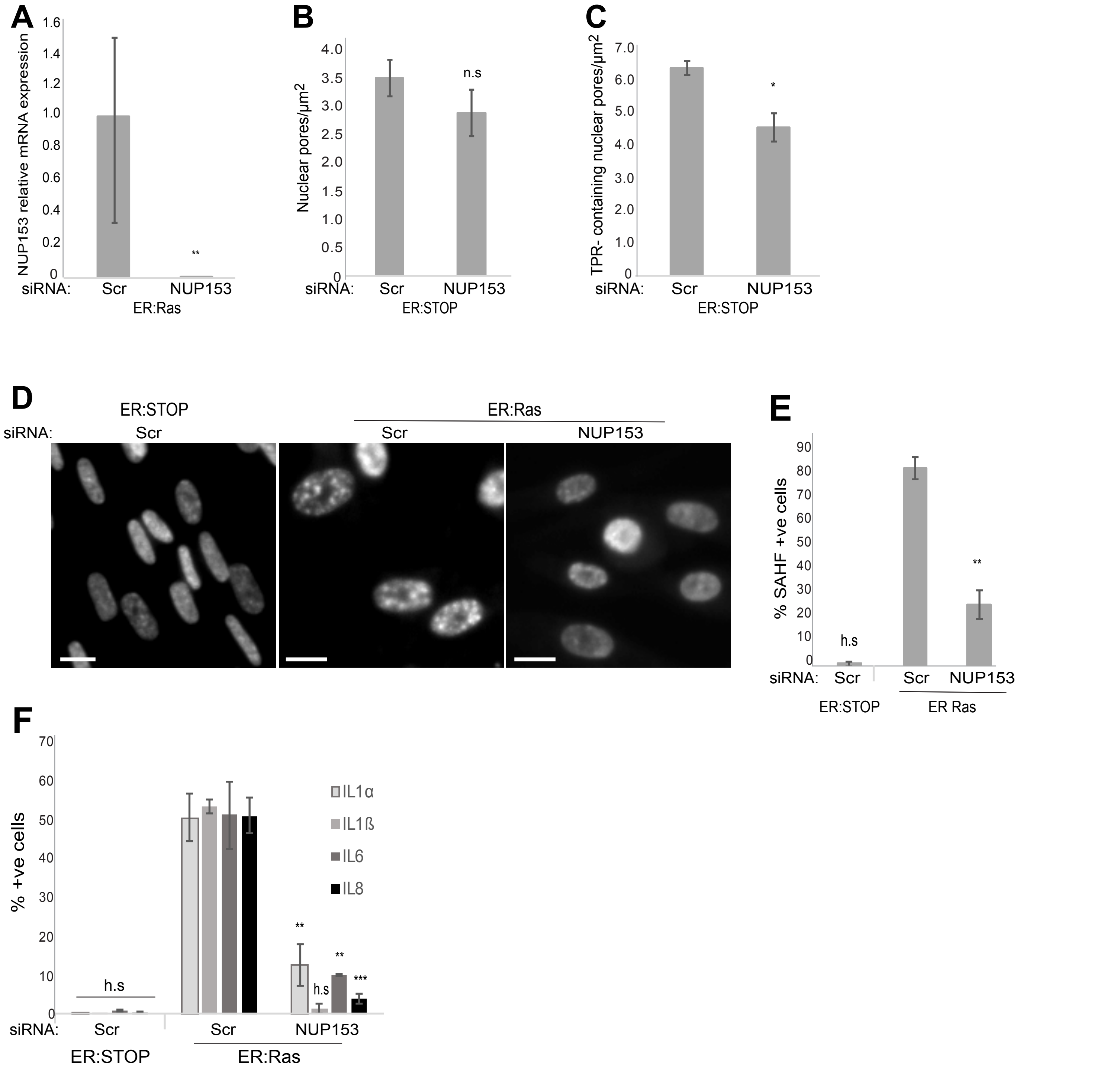

Figure S8 - NUP153 depletion recapitulates TPR depletion phenotype

A) Mean (± SEM) mRNA level, established by qRT-PCR, for NUP153, in 4HT-treated ER:Ras cells after knockdown with scramble (Scr) or NUP153 siRNAs. Expression is shown relative to ER:Ras cells transfected with Scr siRNAs. Data from 3 independent experiments. **p<0.001

B) Mean (+/- SEM) nuclear pore density (pores/µm^2) in 4HT treated ER-Stop cells after knockdown with scrambled (Scr) and NUP153 siRNAs as counted by MAB414 staining in 3 independent biological replicates, n.s=non significant.

C) As for B) but for TPR staining, *p<0.01.

D) DAPI staining of non-senescent 4HT treated ER:Stop and OIS (ER:Ras) cells after knockdown with scrambled (Scr) or NUP153 siRNAs (siNUP153). Scale bars 10µm.

E) Mean (+/- SEM) % of cells containing SAHF in 4HT-treated ER:Stop and ER:Ras cells after knockdown with scramble (Scr) siRNAs and in 4HT-treated ER Ras cells with NUP153 siRNAs. Data from 3 independent experiments. **p<0.01, h.s=highly significant.

F) Mean (± SEM) % of cells positive for SASP cytokines (IL1α, IL1β, IL6,IL8) measured by immunostaining in 4HT-treated ER:Stop and ER:Ras cells after knockdown with scramble (Scr) siRNAs and in 4HT-treated ER:Ras cells with NUP153 siRNAs. Data from 3 independent experiments. **p<0.01, ***p<0.001, h.s=highly significant.

Table S1- siRNAs

Name	Type	Reference
SCR	on targeting plus non targeting control	D-001810-10-59
TPR	on target plus smart pool	L-010548-00
TPR-6	on target plus	J-010548-06
TPR-7	on target plus	J-010548-07
TPR-8	on target plus	J-010548-08
TPR-9	on target plus	J-010548-09
POM121	on target plus smart pool	L-017575-04
ASF1a	on target plus smart pool	L-020222-02-0020
NUP153	on target plus smart pool	L-005283-00-0010

Table S2 - antibodies

Name	Company	reference	application and dilution
TPR	Abcam	ab84516	WB (1:200), IF (1:500)
Pom121	Millipore	AB6041	WB (1:500), IF (1:200)
Actin	Santa Cruz Biotechnology	sc-1616	WB (1:2000)
MAB414	Abcam	ab24609	IF (1:50)
LaminB	Santa Cruz Biotechnology	sc-6216	IF (1:1000)

Table S3- Primers

Name	Sequence
Pom121-Fw	TTCAACGTGAGCAGCACAAC
Pom121-Rev	CAAAAGTGTTGCCGAAAGGTG
TPR-Fw	CTGAAGCAATTCATTCGCCG
TPR-Rev	GGCATATCTTCAGGTGGCCC
ASF1a-Fw	CAGATGCAGATGCAGTAGGC
ASF1a-Rev	CCTGGGATTAGATGCCAAAA
Actin-Fw	CATGTACGTTGCTATCCAGGC
Actin-Rev	CTCCTTAATGTCACGCACGAT
IL1α-Fw	AGTGCTGCTGAAGGAGATGCCTGA
IL1α-Rev	CCCCTGCCAAGCACACCCAGTA
IL1β-Fw	TGCACGCTCCGGGACTCACA
IL1β-Rev	CATGGAGAACACCACTTGTTGCTCC
IL6-Fw	CCAGGAGCCCAGCTATGAAC
IL6-Rev	CCCAGGGAGAAGGCAACTG
IL8-Fw	GAGTGGACCACACTGCGCCA
IL8-Rev	TCCACAACCCTCTGCACCCAGT
NUP153-Fw	CAGGCCAAAAGAGAAAAGGTGG
NUP153-Rev	GCGAAACCAGGGCTTTTCAG
